# Supplementary material for: Rhythmic tapping difficulties in adults who stutter: A deficit in beat perception, motor execution, or sensorimotor integration?
Source: PLoS One. 2023 Feb 3;18(2):e0276691. doi: 10.1371/journal.pone.0276691 (PMC9897587; doi:10.1371/journal.pone.0276691)
Supplement: S1 File — S1 Fig. (a) Correlation between this average Tapping Asynchrony in the condition REACT (i.e. the average finger Reaction Time) and the SSI score of PWS. (b) Correlation between this reaction time variability and the SSI score of PWS. S2 Fig. Tapping Force Variability in the reproduction task of an isochronous pattern, after passive listening (ISO_REPRO) and in both tasksof synhcronization to a 4-beat metered isochronous pattern (1:1_ISO_SYNC) or to a non-isochronous pattern (NONISO_SYNC). S3 Fig. (a) Correlation between the average log(CV) value and SSI score of PWS. (b) Correlation between the average log(CV) and the Reaction Time Variability (in the condition REACT) of each participant (N = 32). (c) Correlation between log(CV) and the Tapping Force Variability on each train of taps produced in the condition ISO_REPRO. S4 Fig. (a) Correlation between the average PE and the SSI score of PWS. (b) Correlation between the average PE in the condition ISO_REPRO, and the average Reaction Time in the condition REACT of each participant (N = 32). S5 Fig. (a) Correlation between the average PA in the condition 1:1_ISO_SYNC, and the SSI score of PWS. (b) Correlation between the average log(PLV) vaalue in the condition 1:1_ISO_SYNC, and the SSI score of PWS. S6 Fig. (a) Correlation between the average Phase Angle (PA) in the condition 1:1_ISO_SYNC, and the average Reaction Time in the condition REACT of each participant (N = 32). (b) Correlation between the average Phase Locking Value (logit((PLV)) in the condition 1:1_ISO_SYNC, and the average Variability in Reaction Time in the condition REACT of each participant. S7 Fig. (a) Correlation between the Phase Angle (PA) and the Tapping Force (TF) of each tap in the conditions 1:1_ISO_SYNC and 1:4_ISO_SYNC. (b) Correlation between the logit(PLV))value and the Tapping Force Variability on each train of taps produced in the conditions 1:1_ISO_SYNC and 1:4_ISO_SYNC. People who stutter (PWS, N = 16) are compared to with matched contr [file pone.0276691.s001.pdf]

A. ASSESMENT of MUSICAL TRAINING

"Musical training" was auto-evaluated on a 3-levels scale : 2 – Indicated a high level of experience, meaning that the participant had been playing a music instrument for more than 5 years seriously, with a regular current practice; 1 – Indicating a level of moderate experience, meaning that the participant played a musical instrument less than 5 years, and/or did not practice intensively; 0 – No experience, meaning that the person never practiced an instrument on a regular basis. Musical Training was only used, like Gender and Age, as an individual factor for matching participants in both groups of PNS and PWS.

B. COMPLEMENTARY RESULTS

B.1 Motor delays and variability

B.1.1 Correlation of RT and RT_Var with stuttering severity

No significant correlation was observed between the SSI score of each PWS and the log value of his/her average Reaction Times (Pearson's correlation: r(14)=0.41, p=.11)(see Figure B1a) or its variability (r(14)=0.40, p=.12)(see Figure B1b).

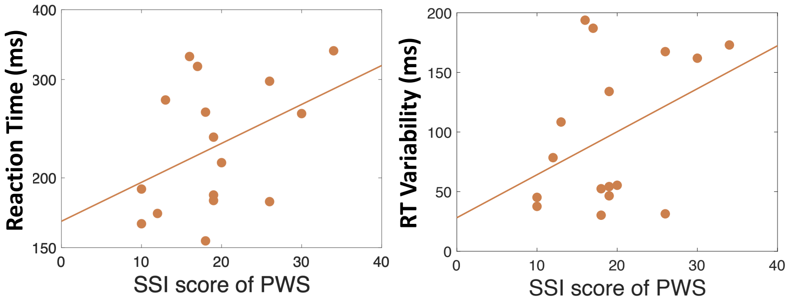

S1 Fig. (a) Correlation between this average Tapping Asynchrony in the condition REACT (i.e. the average finger Reaction Time) and the SSI score of PWS. (b) Correlation between this reaction time variability and the SSI score of PWS.

B.1.2 Variability in Tapping Force

The variations of TF_Var were explored, considering the mixed model [TF_Var ~ GROUP * CONDITION+ 1|Participant], with CONDITION = {ISO_REPRO ; 1:1_ISO_SYNC ; NONISO_SYNC].

No significant difference in tapping force variability was observed between PNS and PWS in the two tasks ISO_REPRO (ΔTF_Var PNS-PWS = 0.008 ± 0.021 a.u, z=0.37, p=.71) and 1:1_ISO_SYNC (ΔTF_Var PNS-PWS = -0.005 ± 0.018 a.u, z=-0.25, p=.80), in which participants were reproducing an isochronous pattern on their own, or tapping in synchrony with it (see Figures B2a and B2b). In the task NONISO_SYNC, however, when participants tapped along a non-isochronous pattern, a significantly greater variability in tapping force was observed for PWS, compared to PNS (ΔTF_Var PNS-PWS = 0.040 ± 0.019 a.u, z=1.90, p=.031) (see Figure B2c).

The lowest level of Tapping Force variability was observed in the "simple" synchronization task 1:1_ISO_SYNC, with a significant increase in ISO_REPRO (ΔTF_Var ISO_REPRO-1:1_ISO_SYNC = 0.035 ± 0.009 a.u, z=3.79, p=.0003), and even more in the "complex" synchronization task NONISO_SYNC (ΔTF_Var INONISO_SYNC-1:1_ISO_SYNC = 0.036 ± 0.007 a.u, z=4.90, p<.0001) (see Figure B2).

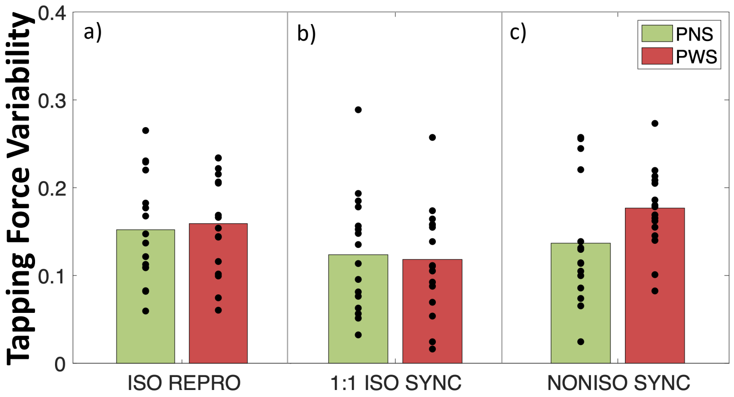

S2 Fig. Tapping Force Variability in the reproduction task of an isochronous pattern, after passive listening (ISO_REPRO) and in both tasksof synhcronization to a 4-beat metered isochronous pattern (1:1_ISO_SYNC) or to a non-isochronous pattern (NONISO_SYNC).

B.2 Ability to perceive and reproduce periodicity

B.2.1 Global acceleration or slowdown in the condition ISO_REPRO

A first mixed model [ITI ~ TapNumber*GROUP + 1|Participant] was considered over the first 24 taps in the condition ISO_REPRO.

In this condition, during which the participants listened passively to an isochronous pattern, and then reproduced it on their own (i.e. without the help of external auditory triggers), no systematic drift of ITI over time was observed: no significant correlation was found between the ITI values and the tap number in the train (from 1 to 24) (slope: -0.22 ± 0.13 ms/tap, p=.078, R=-0.53; these values correspond to an average acceleration of about 1% after 24 taps, which can be considered as negligible). This absence of significant acceleration or slowdown was comparable for both PWS and PNS (GROUP effect: df=1, LRatio=1.23, p=.27).

B.2.2 Correlation of CV in ISO_REPRO, with stuttering severity and other indices of motor variability

For PWS, no significant correlation between their SSI score and their average log(CV) value was observed for both the stabilized part (taps 9 to 24) and the first 8 taps of the condition ISO_REPRO (Pearson's correlation : r(14)=0.27, p=.30 and r(14)=0.21, p=.44, respectively)(see Figure B3a).

No significant correlation was also observed, for the whole group of participants, between the average log(CV) value of each individual, over the stabilized part (taps 9 to 24) of the condition ISO_REPRO, and his/her variability in Reaction Time (RT_Var) in the REACT condition (Pearson's correlation : r(30)=0.19, p=.29). This correlation did not depend on the GROUP (df=1, LRatio=0.038, p=.35)(see Figure B3b).

No significant correlation was also observed between log(CV) and the Tapping Force variability (TF_Var) of each train of taps produced over the stabilized part (taps 9 to 24) of the condition ISO_REPRO (Repeated measures correlation: r(33)=-0.02, p=.90), regardless of the group (df=1, LRatio= 0.79, p=.37), as well as over the very first taps (R(33)=0.09, p=.60), again regardless of the group (df=1, LRatio= 1.92, p=.17) (see Figure B3c).

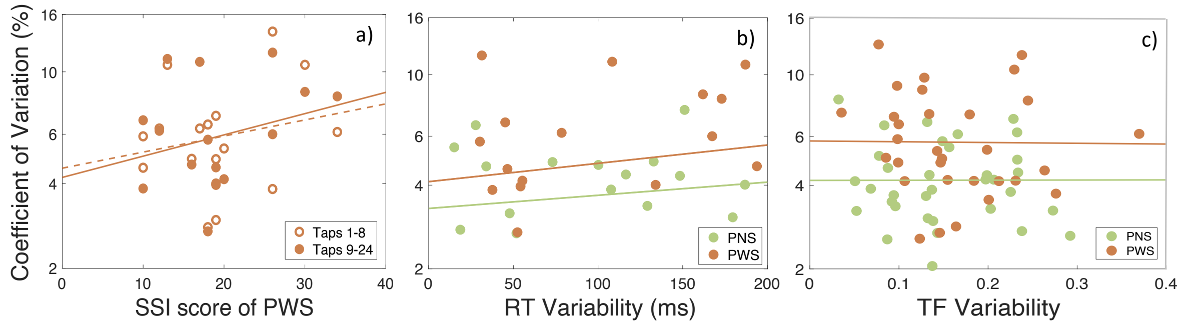

S3 Fig. (a) Correlation between the average log(CV) value and SSI score of PWS. (b) Correlation between the average log(CV) and the Reaction Time Variability (in the condition REACT) of each participant (N=32). (c) Correlation between log(CV) and the Tapping Force Variability on each train of taps produced in the condition ISO_REPRO.

B.2.3 Correlation of PE in ISO_REPRO, with stuttering severity and other indices of motor delays

No significant correlation was observed between the SSI score of PWS and their average Periodicity Error during the stabilized part (taps 9 to 24) of the condition ISO_REPRO (Pearson's correlation: r(14)=0.05, p=.85), or during the first part (taps 1 to 8) (r(14)=0.29, p=.28) (see Figure B4a).

No significant correlation was also observed between the average Tapping Asynchrony of each individual in the REACT condition (i.e. the average Finger Reaction Time), and his/her average PE, during the stabilized part (taps 9 to 24) of the condition ISO_REPRO (Pearson's correlation: r(30)=0.28, p=.13). This correlation did not depend on the GROUP (df=1, LRatio=0.0004, p=.98)(see Figure B4b).

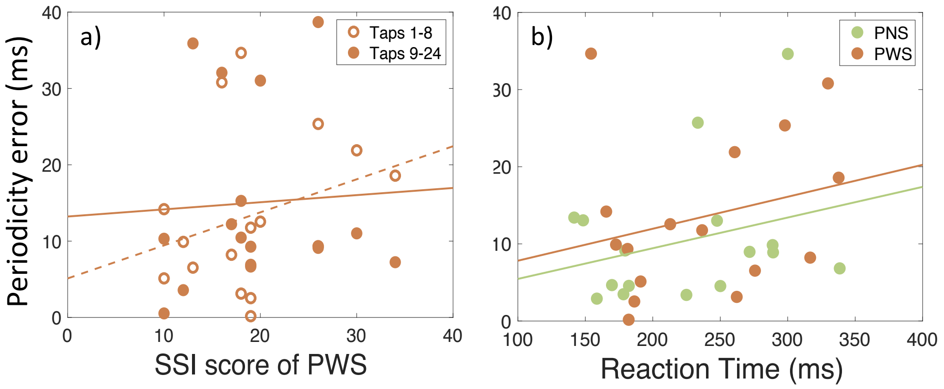

S4 Fig. (a) Correlation between the average PE and the SSI score of PWS. (b) Correlation between the average PE in the condition ISO_REPRO, and the average Reaction Time in the condition REACT of each participant (N=32).

B.3 Synchronization abilities: Phase Angle (accuracy), and Phase Locking Value (consistency)

B.3.1 Stuttering severity

No significant correlation was found between the SSI score of PWS and their average synchronization accuracy (PA) or consistency (PLV) in the condition 1:1_ISO_SYNC (Angular-linear correlation: r(14)=0.20, p=.72, see Figure B5a, and Pearson's correlation: r(14)=0.08, p= .75, see Figure B5b, respectively).

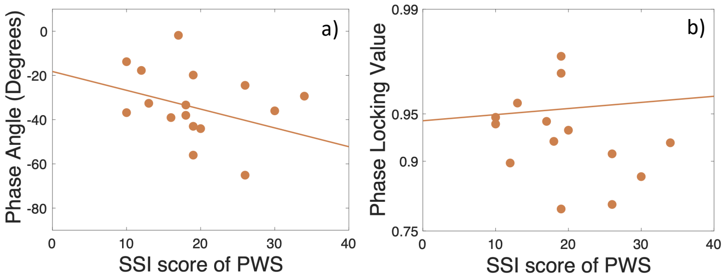

S5 Fig. (a) Correlation between the average PA in the condition 1:1_ISO_SYNC, and the SSI score of PWS. (b) Correlation between the average log(PLV) vaalue in the condition 1:1_ISO_SYNC, and the SSI score of PWS.

B.3.2 Correlation with RT and RT_Var

No significant correlation was observed in the PNS group or the PWS one, between the average Reaction Time of each individual in the condition REACT, and his/her average degree of NMA over the stabilized part (taps 9 to 24) of the condition 1:1_ISO_SYNC (angular-linear correlation: r(30)=0.12, p=.90 in PNS; r(30)=0.58, p=.065 in PWS) (see Figure B6a).

No significant correlation was also observed between the logit(PLV) value of each individual over the stabilized part (taps 9 to 24) of the condition 1:1_ISO_SYNC, and of his/her average variability in Reaction Time (RT_Var) in the REACT condition (Pearson's correlation: r(30)=-0.24, p=.19)(see Figure B6b). This correlation did not depend on GROUP (df=1, LRatio = 0.43, p=.52 for RT_Var).

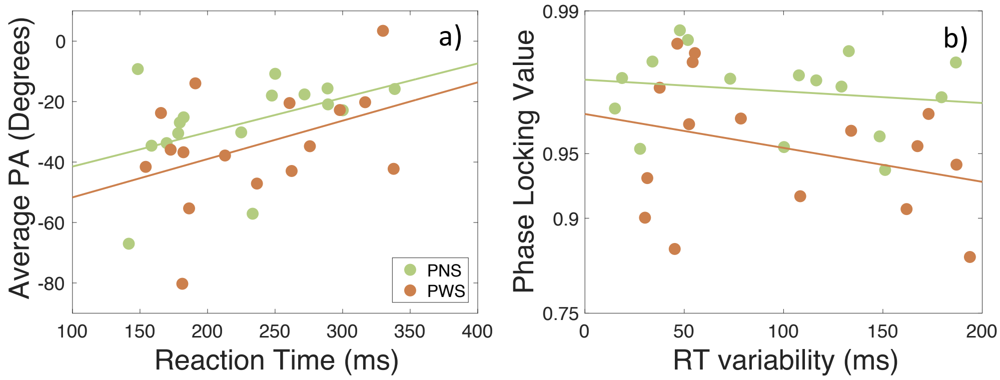

S6 Fig. (a) Correlation between the average Phase Angle (PA) in the condition 1:1_ISO_SYNC, and the average Reaction Time in the condition REACT of each participant (N=32). (b) Correlation between the average Phase Locking Value (logit((PLV)) in the condition 1:1_ISO_SYNC, and the average Variability in Reaction Time in the condition REACT of each participant.

B.3.3 Correlation with TF and TF_Var

The Bayesian circular mixed model [PA ~ TF *GROUP* CONDITION + 1|Participant] with CONDITION = {1:1_ISO_SYNC ; 1:4_ISO_SYNC ; NONISO_SYNC} was considered to test the "sensory accumulation theory" (62) that predicts that the degree of Negative Mean Asynchrony is related to the Tapping Force. The correlation coefficient between both variables was computed for each group and each condition, using a repeated measures correlation test (R package rmcorr).

Within each condition, variations in PA and TF may especially reflect the effect of Beat Strength. No significant correlation of PA with TF was observed within the condition 1:1_ISO_SYNC (Repeated measures correlation: r(903)= 0.03, p=.39), regardless of the GROUP (df=1, LRatio= 2.73, p=.099). In NONISO_SYNC, a significantly negative correlation was observed for PWS (r(461)=-0.16, p=.0004), whereas no significant correlation was observed for PNS (r(451)=0.02,

p=.60). No significant correlation was found between the two parameters, when considering altogether the taps produced in both conditions 1:1_ISO_SYNC and NONISO_SYNC (r(1949)= -0.01, p=.58)., which may rather reflect the variations in PA and TF induced by rhythmic complexity and pulse doubling. This was the case in both groups (df=1, LRatio=2.84, p=.092) (see Figure B7a).

As concerns the variations of logit(PLV) and TF_Var, no significant correlation was observed between the two parameters within each condition 1:1_ISO_SYNC (r(90)=-0.03, p=.78) and NONISO_SYNC (r(113)=-0.23, p=.15), considered separately, without significant interaction with the group (df=1, LRatio= 0.47, p=.49 for 1:1_ISO_SYNC; LRatio=0.43, p=.51 for NONISO_SYNC). However, a significant correlation was observed over their gathered data (r(236)= -0.14, p=0.027), regardless of GROUP (df=1, LRatio=0.012, p=.91) (see Figure B7b).

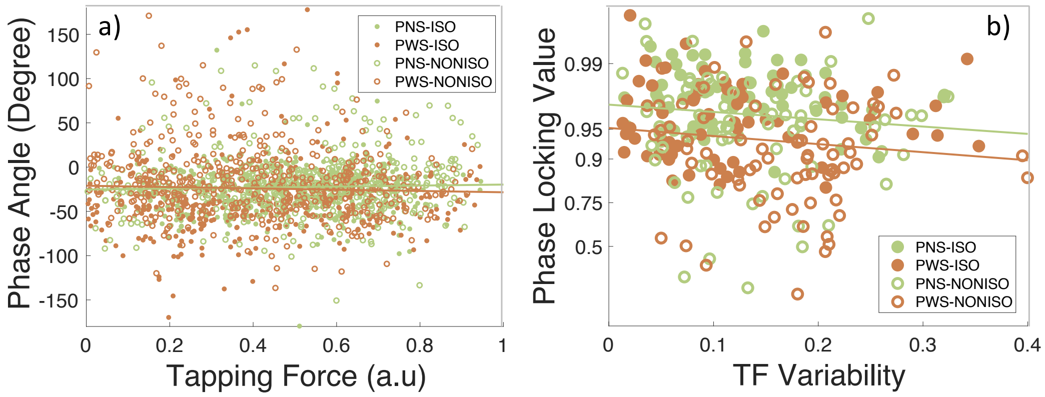

S7 Fig. (a) Correlation between the Phase Angle (PA) and the Tapping Force (TF) of each tap in the conditions 1:1_ISO_SYNC and 1:4_ISO_SYNC. (b) Correlation between the logit(PLV))value and the Tapping Force Variability on each train of taps produced in the conditions 1:1_ISO_SYNC and 1:4_ISO_SYNC. People who stutter (PWS, N=16) are compared to with matched control particpants without speech disorders (PNS, N=16)

B.3.4 Effect of rhythmic complexity (non-isochrony and pulse doubling)

During NONISO_SYNC, taps falling on strong beats were realized with lower PA values (i.e., smaller NMA), and thus improved accuracy, compared to 1:1_ISO_SYNC (ΔPANONISO-ISO= -8.3 ± 2.2 degrees, HPD=[-12.7 -3.9]) (see Figures 6a and 6c of the main article). A similar improvement in accuracy was also observed for the taps falling on weak beat (ΔPANONISO-ISO= -9.2 ± 1.5 degrees, HPD=[-12.2 -6.5]) and this effect of rhythmic complexity did not depend on the group.

On the contrary, the average PLV (synchronization consistency) was significantly lower in NONISO_SYNC, compared to 1:1_ISO_SYNC, for taps synchronized with strong beats (Δlogit(PLV)NONISO-ISO = -0.35 ± 0.13, z=2.67, p=.008) as well as those synchronized with weak beats (Δlogit(PLV)NONISO-ISO =-0.24 ± 0.12, z=-2.10, p=.041) (see Figures 7a and 7c of the main article). Again, this effect of rhythmic complexity was similar in both groups.

Finally, TF in the condition NONISO_SYNC did not vary significantly, compared to the condition 1:1_ISO_SYNC for the PNS (ΔTF NONISO_SYNC-1:1_ISO_SYNC = 0.029 ± 0.016 a.u., z=1.83, p<.0002), or the PWS (ΔTF NONISO_SYNC-1:1_ISO_SYNC = -0.015 ± 0.015 a.u, z=-0.99, p=.54) (see Figures 5a and 5c).
