# Supplementary material for: Rhythmic tapping difficulties in adults who stutter: A deficit in beat perception, motor execution, or sensorimotor integration?
Source: PLoS One. 2023 Feb 3;18(2):e0276691. doi: 10.1371/journal.pone.0276691 (PMC9897587; doi:10.1371/journal.pone.0276691)
Supplement: Attachment — Submitted filename: Rebuttal_270922.pdf [file pone.0276691.s003.pdf]

Rebuttal letter

Reviewer #2: The current manuscript revision is greatly improved and easier to follow, and the discussion in particular is a lot clearer. It was also nice that the results were condensed to the most interesting, but also that the full analyses were reported in supplementary material. The result structure was easier to follow since it has been condensed, but it was still a bit tricky to track which task and which dependent variable was being analysed. It helped to have the models written out.

Perhaps the authors could consider having a more systematic labelling system of headings. At the moment, there are changes in structure, e.g., levels of headings, bolding etc, which make it difficult to clearly see the patterns.

> *We worked on this, so that there are now 3 clear levels of headings. A 4 fourth level of organization is sometimes marked by bullet points.*

Some of the writing is sometimes a bit unclear as well and could be edited further.

> *The entire manuscript has been edited once again by a native speaker of English.*

But overall, the paper is getting into good shape, and the authors have done a good job condensing a lot of dependent variables into a digestible manuscript.
Here are some minor comments to improve clarity:
Pg. 4, lines 64-65: "some other studies" are mentioned, but only one is cited.

> *This was replaced by 'another study'*

Pg. 7: "the observation that steady state-evoked potentials appear in the delta frequency range in subjects who were passively listening to a rhythmic sequence at 2.4Hz provides strong support to this hypothesis". The existence of steady-state evoked potentials could actually be based on populations of neurons firing in synchrony, not necessarily that they are reflecting the entrainment on endogenous oscillations. There is a big debate about this in the field, so it's important to clarify this point. E.g., see Zoefel, ten Oever & Sack, 2019: Neural oscillations in the processing of rhythmic input: More than a regular repetition of evoked neural responses. Frontiers in Neuroscience.

> *We agree with this comment and this paragraph on L111-115 was re-worded as;*
>
> *"Although there is still ongoing debate on this endogenous oscillator entrainment hypothesis (49,50), the observation that steady state-evoked potentials appear in the delta frequency range [0.5 – 4 Hz] in subjects who were passively listening to a rhythmic sequence at 2.4Hz, provides support for this hypothesis (43,44,51)", with*
>
> *[49]. Doelling KB, Assaneo MF, Bevilacqua D, Pesaran B, Poeppel D. An oscillator model better predicts cortical entrainment to music. Proc Natl Acad Sci. 2019;116(20):10113–21.*
>
> *[50]. Zoefel B, Ten Oever S, Sack AT. The involvement of endogenous neural oscillations in the processing of rhythmic input: More than a regular repetition of evoked neural responses. Front Neurosci. 2018;12:95.*

Pg. 10, line 185: the greater negative mean asynchrony can be explained by a weaker tapping force – please explain why. The logic behind this is unclear for the moment.

> *We added an explanation about 10 lines above (L173-177), and clarified the sensory accumulation theory: "In addition to slower processing and integration, this so-called "sensory accumulation" theory further predicts that the magnitude of auditory-tactile delay, and the resulting NMA, depends on stimulation intensity, which, in case of tapping, is hypothesized to concern the tapping force. The NMA is therefore hypothesized to decrease when tactile-kinesthetic feedback in the form of tapping force increases"*

We hope that we addressed the confusion re. the logic behind that sentence on L183 "the larger degree of NMA observed in PWS can be explained by a weaker tapping force, as predicted by the sensory accumulation theory".

Table 1: musical training is listed as 0, 1, 2 for PWS and as no/yes for PNS – these should be the same scale.

The PNS and PWS were matched in musical training. The notation is modified in the table, accordingly.

Figure 1: For 1:4_ISO_SYNCH – were the 1st and 4th beats in the example stimuli accented? If so, this should be made clear in the diagram with e.g., an accent marker.

In that condition, the first and fifth beats were not "accentuated" but were the only ones marked with an external stimulus.

We explained this on P15, 271-272, in the description of the task 1:4_ISO_SYNC, as follows: "the external auditory stimuli were played back every 4 beats only – on the 1st and the 5th beats of the 8-beat cycle".

In addition, this is also illustrated in the recapitulative Figure 1.

Pg. 16: Only the 9th and 24th taps were considered for analysis. Why? Please motivate the reason for this in the text.

We did not consider the 9th and 24th taps, but the taps number 9 to 24, i.e., the 2nd and 3rd 8-beat cycles of taps, taken as the "stabilized" phase of the task. It was hypothesized that some sensorimotor adaptation and learning might occur during the first cycle of 8 taps and carried over to the remainder of the trial.

The word "Taps 9-24" was replaced by "Taps 9 to 24" to avoid confusion

Table 2 is helpful for following the measures taken across the different tasks.

We are happy that it improved the paper.

Figure 3 caption seems to be switched around, with 3a as periodicity in the caption, but coefficient of variation in the figure.

Modified

Pg. 27 – why was the Bayesian model Group + Time and no interaction?

*The notation in the bpnreg package (for circular mixed models) is actually different from that of the nlme package (for linear mixed models). In the package bpnreg, the notation "Group + Time" actually considers the possible interaction Group*Time between both factors. We refer the readers to Cremers et al. (2017) for more details about the use of the bpnreg package.*

Pg. 27, line 512: "however, no significant difference…" – I don't think you need a "however" here. You could just say "there was no significant difference…".

The word "however" has been removed.

Pg. 33, line 645 – after explaining all the results across the various measures, it might be nice in this first paragraph to more clearly link the theoretical side with the tasks measured. E.g., 1- in the execution of movements (as measured in xx task/s).

This paragraph, L622-628, has been modified as follows:

“The study investigated the rhythmic tapping behavior of people who stutter compared to people who do not stutter and considered several levels of processing at which differences were hypothesized to occur: 1- the execution of movements, in particular their initiation (as measured in the task REACT), 2- the perception of beat, at a given periodicity (as measured in the task ISO_REPRO), 3- the on-line adaptation and improvement of their accuracy and consistency, based on sensory feedback (as measured in the tasks 1:1_ISO_SYNC, 1:4_ISO_SYNC and NONISO_SYNC).”

Pg. 36, Lines 739-743: this sentence is very long and refers to already presented information. This could be written more concisely to avoid having to refer to information “already stated above”.

The sentence on L712-713 has been modified as follows:

“Several arguments were provided in the preceding section (4.1) that exclude the idea that timing differences between PWS and PNS simply result from an impaired motor execution.”

Pg. 40, line 812, do you mean central nervous system?

Thank you for pointing this out. It has been corrected accordingly.

Pg. 41, line 835: do you mean *decreased* PLV in PWS?

Yes., thank you for noticing the mistake. This has been corrected.
